# A new species of psallopinous plant bug from the Malay Peninsula (Heteroptera, Miridae, Psallopinae)

**DOI:** 10.3897/zookeys.679.13130

**Published:** 2017-06-08

**Authors:** Aleksander Herczek, Yuri A. Popov, Jacek Gorczyca

**Affiliations:** 1 University of Silesia in Katowice, Department of Zoology, 40-Katowice, Bankowa 9, Poland; 2 Borissiak Paleontological Institute Russian Academy of Sciences, Profsoyuznaya Str. 123, 117997 Moscow, Russia, † deceased

**Keywords:** Heteroptera, Miridae, new species, Psallopinae, *Psallops*, Southern Asia

## Abstract

The paper presents description and illustrations of a new peculiar species from the genus *Psallops*, *P. coloratus* **sp. n.** from Southeast Asia (Singapore). Photographs, line drawings of the general habitus and a short comparison with a species from Thailand are provided.

## Introduction

Schuh (1976) established a new subfamily Psallopinae (Miridae) for the single member *Psallops ocullatus* Usinger, 1946. Among the main characters of *Psallops* the author listed anterior margin of pronotum slightly bent inwards, 2–segmented tarsi, subapical tooth on the tarsal claw, bristle–like parempodia, phallotheca fused with the phallobase, a simple form of vesical. Additionally, many authors consider the presence of nine metafemoral trichobothria, the head rounded in frontal view, and one or two closed cells on the membrane to be characteristic. In our estimation, one of the basic characters of the Psallopinae is the presence of enlarged eyes that are exceptionally well–developed dorso–ventrally and almost touch each other reaching the pharynx (Herczek and Popov 2014). However, it should be noted that these features are not unique for *Psallopinae*. They appear in some other subfamilies among the Miridae (e.g., *Isometopinae*, *Cylapine* or *Phylinae*) although some of these features are not fixed within *Psallopinae*. This is the main difficulty in providing a clear, morphological definition of this subfamily. Therefore, most often we rely on a combination of morphological features. Konstantinov (2003) analysed in detail the male genitalia in *Psallopinae* based on two species, one of which also came from Singapore (*Psallops* sp. no. 1). According to this author, it is premature to draw any taxonomic conclusion because too little material has been examined so far. The morphology of genitalia shows many plesiomorphic features and cannot be used to delineate the systematic position of the group. Having agreed with this statement, we should not underestimate the usefulness of such characters in the determination of species (despite the fact that species variations for these structures are still poorly examined) . After comparing the specimens described so far, we have noticed clear differences in the construction of left paramere (despite the constancy with the overall schema) and aedeagus (especially the endosoma).

There are nine species representing the subfamily Psallopinae that are known from the Southeast Asia. The first reports came from Japan where three species were described by Yasunaga (1999), *Psallops myiocephalus* (Nagasaki), *P. nakatanii* (Fanaura) and *P. yaeyamanus* (Yaeyema). The next was Lin’s paper (2004), describing *P. chinensis* (Tijain, China), *P. formosanus* (Nautan, Taiwan), and *P. leeae* (Pintung, Taiwan). *Psallops luteus* (Lin 2006) also comes from Taiwan. Recent reports (Yasunaga et. al. 2010) introduced the Thai species *P. fulvoides* and *P. sakaerat*, which had been caught in the Sakaerat National Environmental Research Station (northeastern Thailand). Among the species listed, four were captured using a light trap, two were found on the trunks of deciduous trees (*Quercus* and fabaceous broadleaf), one was captured in a sweep net, and one in a malaise trap.

## Material and methods

While studying the material in the collection of the Zoologisk Museum Copenhagen, the authors found a specimen of Psallopinae that has not been described to date. This specimen was recorded from Singapore, collected by O. Martin in the Seletar Reservoir and deposited in the collection of the museum. The abdomen was dissected and placed in a separate vial. The parameres and aedeagus were sectioned, immersed in Berlese liquid, and placed on a celluloid board. The board was placed under the specimen. Coloured photographs and drawings were obtained using Nikon Eclipse E 600 microscope and the computer program NIS Elements, Ver. 4.10. Measurements were taken with a micrometre. Classification terminology of the male genitalia follows Konstantinov (2003).

## Taxonomy

### Family Miridae

#### Subfamily Psallopinae

##### Genus Psallops

Taxon classificationAnimaliaHeteropteraMiridae

Usinger, 1946

###### Type species.

*Psallops oculatus* Usinger, 1946; 86.

##### Psallops coloratus sp. n.

Taxon classificationAnimaliaHeteropteraMiridae

http://zoobank.org/7BD9DB14-59E4-419C-B640-E6D0ACE1505C

Figs 1–3 , 4–7

###### Diagnosis.

Pronotum covered with long, strong, protruding black setae; corium with two types of setae: pale, long, semi erect hairs and shorter, dark and adpressed. Second and third antennal segments with long, pale setae, more than twice as segment diameter. Ratio of eye width to vertex width 2.67; ratio of head width to vertex width 6.51; ratio of corium length to cuneus length 4.55.

**Figures 1–3.**
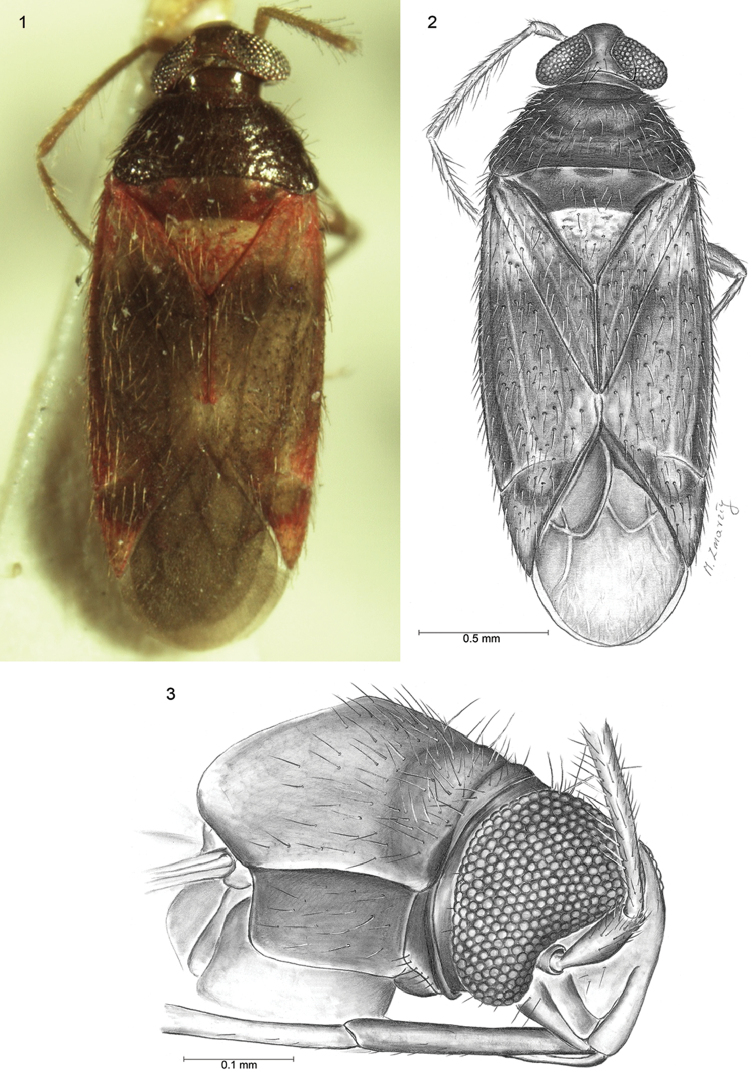
**1, 2** *Psallus coloratus* sp. n. **1** Photograph of dorsal view **2** Drawing of dorsal view **3** Lateral drawing of head. Scale bars: **1, 2** = 0.5 mm; **3** = 0.1 mm.

###### Description.

**Figures 4–7.**
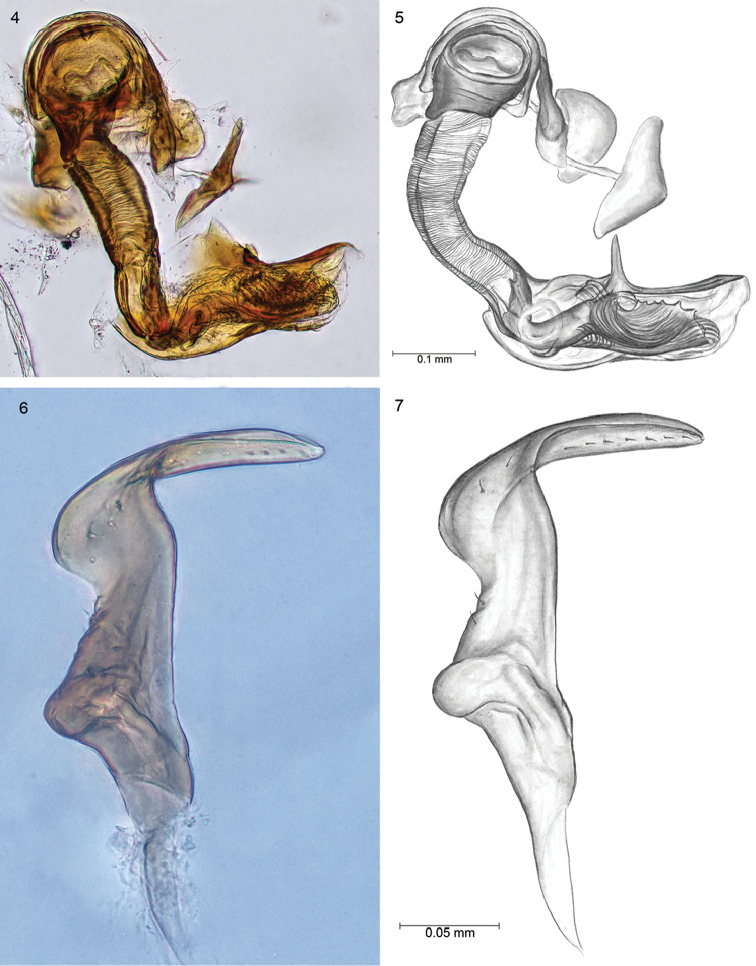
**4, 5** Photograph and drawing of aedeagus and endosoma **6, 7** Photograph and drawing of left paramere. Scale bars 0.1 mm **4, 5**; 0.05 mm **6, 7**.

**Male.** Body elongated, 2.67 long as wide. *Head* 2.33 as wide as long. Eyes large and discoid, occupying nearly entire sides of head, contiguous with anterior margin of pronotum (Figs 1, 2). Vertex not very broad, at the narrowest point 0.37 times as wide as one eye. Clypeus smoothly flush with convex frons; base of clypeus located slightly lower than half eye height; mandibular plate relatively broad, nearly reaching apex of clypeus (Fig. 3). Antennal fossa situated at base of maxillary plate. Antennae four-segmented, segment I shortest, nearly 1/5 length of second; segment III 0.8 times as long as II; segments IV missing. Maxillary plate relatively broad, buccula narrow. *Thorax*: Pronotum without calli with collar-like, flattened anterior margin (Figs 2, 3). Pronotum 1.54 as long as head and 2.22 times wider at basal margin as long. Exposed part of mesoscutum convex, 0.52 times as long as scutellum. Length of mesoscutum and scutellum slightly longer than the length of claval commissure. Corium 4.55 as long as cuneus. Hemelytral membrane with well large developed cell, 2.16 times as long as wide. *Legs*: hind femur approximately 3.6 times longer than maximum width, hind tibia 3.63 times longer than tarsus length. Tarsi two-segmented, second tarsal segment 1.3 times as long as the first; Inner surface of tibia with two rows of bright spines, which length is slightly greater than diameter of tibia. *Male genitalia*: Aedeagus membranous with strong sclerotized dorsal part (Figs 4, 5). Endosoma with complicated, strong sclerotized structure; paramere structure *Lygus*-type (Konstantinov 2003), left paramere scythe-shaped, apical process with seven small teeth. Body of paramere with several short setae. Sensory lobe convex, knee-shaped (Figs 6, 7). Right paramere missing. Head and antennae smoky-yellow with dark back vertex and fuscous clypeus. Eyes silver, labium brown. Pronotum wrinkled, dark brown. Mesoscutum rust, shiny. Apical half part of scutellum reddish, basal part yellowish with a few reddish spots. Basal part of clavus and corium, apex of clavus, external margins of corium and apical 1/2 of cuneus reddish. The remainder of corium brown (just like basal part of cuneus), slightly brighter in middle. Membrane grey dark, with clearly marked cells. Ostriolar peritreme dark-reddish with ivory edge. Median vein distinctly marked. Proepisternum, propleuron and mesoepisternum black-brown. Mesoepisternum reddish brown with ivory back. Metapleurum brown. Fore coxae brown, middle and hind pale yellow; femora brown, weakly thickened, tibia and tarsi yellowish.

**Female.** Uunknown.

###### Material examined.

Holotype: male. Singapore, Seletar Reservoir, 1°24'N, 103°48'E; 7.XI.1991. O. Martin leg., Zoologisk Museum Copenhagen.

###### Measurements

**(in mm).** Male: body length – 2.48; width – 0.93; length of head – 0.24; width – 0.56; height – 0.44; dorsal width of eye – 0.23; width of vertex – 0.09; antennal segments: I – 0.14; II – 0.64; III – 0.52, IV – missing; rostral segments: I – 0.32; II – 0.37; III – 0.34; IV – 0.11 (?); length of pronotum – 0.37; anterior width – 0.40; posterior width – 0.82; length of mesoscutum – 0.16; length of scutellum – 0.31; length of claval commissura – 0.42; length of fore femur – 0.57, width – 0.13; tibia length – 0.62, width – 0.08; tarsus length – 0.23; (I– 0.10, II – 0.16); middle femur length – 0.65, width – 0.14 (tibia and tarsus missing); hind femur length – 0.80, width – 0.22; tibia length – 0.98, width – 0.10; tarsus length – 0.27 (I– 0.11, II – 0.14); length of corium – 1.50; length of cuneus – 0.33; cell length – 0.41; width – 0.19

###### Etymology.

From the Latin *coloratus* (variegated), referring to the different colours of the dorsal surface.

###### Remarks.

The new species is distinguished from the one known from southwestern Asia by a combination of colour features and the construction of a copulatory apparatus. It is distinct in the colour of head (yellow, back of the vertex dark), the third part of the antennae (yellow), the scutellum (1/2 apical part reddish, the basal part yellowish with the reddish spots) and the colour of the legs (forecoxa brown, the middle and the hind pale yellow; femora brown, tibia and tarsus pale yellow). *P. coloratus* sp. n. is by colour related to *Psallops formosanus* Lin, but scutellum, the middle part of corium with clavus and the basal part of cuneus are different. On the other hand, the colour of pronotum is similar to that in *P. nakatani*, *P. ponapensis* and *P. yaeyamanus*. In turn, the colour of head shows affinities with that found in *P. sakaerat*, and antennal segments I , II are coloured like those in *P. yaeyamanus*. The colour of mesoscutum is similar to the one described in *P. leeae*, *P. formosanus* and *P. yapensis*.

Some metric features decisively distinguish *P. coloratus* from the species known from the Southeast Asia regions. These are: the ratio of the eye width to the vertex width (2.67), the head width to the vertex width (6.51), antennal segment II length to the pronotum width (1.73), and others. The construction of left paramere is also different. Although the shape of the lob sensor is reminiscent of the one observed in *Psallops sakaerat* Yasunaga, the paramere body and the apical process are developed differently. Additionally, a vast difference is observed in the construction of the aedeagus, which is characterised by a complex of highly sclerotized structures in the endosoma (Figs 4, 5).

## Supplementary Material

XML Treatment for Psallops

XML Treatment for Psallops coloratus
